# Gastric Foveolar Hyperplastic Polyps in 2 Children With Short Bowel Syndrome on Long-Term Teduglutide

**DOI:** 10.1097/PG9.0000000000000389

**Published:** 2023-11-13

**Authors:** Jonathan A. Salazar, Jeffrey D. Goldsmith, Lissette Jimenez, Victor L. Fox, Christopher P. Duggan, Alexandra N. Carey

**Affiliations:** From the * Division of Gastroenterology, Hepatology and Nutrition, Boston Children’s Hospital, Harvard Medical School, Boston, MA; † Department of Pathology, Boston Children’s Hospital, Harvard Medical School, Boston, MA.

**Keywords:** intestinal failure, parenteral nutrition, teduglutide, short bowel syndrome, foveolar hyperplastic polyps

## Abstract

The natural history of short bowel syndrome involves intestinal adaptation wherein the remnant small intestine undergoes histologic and anatomic changes aimed at increasing absorption. Teduglutide—a glucagon-like peptide 2 analog approved for pediatric use in 2019—stimulates this process by causing proliferation of intestinal epithelial cells resulting in increased villous height and crypt depth. Food and Drug Administration approval for pediatric patients followed safety and efficacy studies in children that were limited to 24-week duration. Pediatric-specific postmarketing studies evaluating long-term safety and efficacy are underway. Formation of colorectal polyps has been repeatedly observed in studies of adult patients on long-term teduglutide, including in individuals without endoscopic evidence of polyps before treatment initiation. Recent studies, however, suggest increased risk of small bowel hyperplastic and dysplastic polyp formation with long-term glucagon-like peptide 2 analog use. We report 2 cases of small bowel foveolar hyperplastic polyps found during surveillance endoscopies after 1 year of treatment with teduglutide.

## INTRODUCTION

Intestinal failure (IF) is defined as a reduction in gastrointestinal function below what is necessary for digestion and absorption for adequate nutrient, fluid, and growth requirements (1). IF secondary to short bowel syndrome (SBS) is a result of either surgical or congenital defects that decrease the total mass and absorptive capacity of the gastrointestinal system, often necessitating long-term parenteral support (2,3). The remaining intestine undergoes a functional and structural compensatory process increasing the absorptive capacity of the remaining bowel. Medical therapies have been aimed at intestinal rehabilitation by managing the complex sequelae of SBS (2,4,5).

With the advent of teduglutide, a glucagon-like peptide 2 (GLP2) hormone analog, a novel pharmacologic option became available (6). GLP2 is an enteroendocrine hormone produced in the terminal ileum and proximal colon. In addition to increasing portal blood flow, regulating gastric motility and acid secretion, animal models also show that GLP2 causes crypt cell proliferation and decreases enterocyte apoptosis, leading to increased surface epithelium and improved nutrient absorption (7). Teduglutide similarly increased villus height, crypt depth, and enterocyte proliferation in adult patients with SBS (8). A 24-week phase III pediatric clinical trial demonstrated both the safety and efficacy of teduglutide use in pediatric patients with IF due to SBS (9).

The development of primarily colorectal polyps as a sequela of teduglutide was suggested in post hoc analysis of adult trials and in a pooled safety analysis. In the Randomized control trial/extension study, small bowel polyps were found in 2 out of 173 patients (1.2%) and colonic polyps were found in 5 out of 173 patients (2.9%) (10). In the post hoc analysis, 12% (9/73) of patients had colonic polyps before teduglutide initiation and 18% (9/50) had colonic polyps at postexposure colonoscopies, though limited medical history, polyp risk factors, and histology were available (11). A recent case report noted the development of several adenomatous polyps in the duodenum and jejunum in an adult treated with teduglutide for 41 months (12). Though neither baseline upper endoscopy nor colonoscopy was required in the trials of pediatric patients, no evidence of small bowel polyp development was identified in postexposure colonoscopy (9). In a pooled analysis of 89 patients, 1 cecal polyp was identified and no evidence of neoplasia was found (13). In a recent retrospective review of 35 patients with SBS, however, polypoid lesions were found in 10 out of 35 patients with a mean teduglutide use of 23 months. Of these 10 lesions, 8 were found in the small bowel (14).

In accordance with the manufacturer recommendations label, a colonoscopy is performed before treatment initiation if screening fecal occult blood testing is positive and at 1 year after treatment initiation. Upper endoscopy is performed if clinically indicated. In the 2 cases reported here, patients underwent colonoscopy after 1 year of treatment per the manufacturer recommendation. Upper endoscopies were performed in both cases based on clinical need. We report 2 cases of foveolar hyperplastic polyps in the upper intestinal tract after 1 year of treatment with teduglutide.

## CASE PRESENTATIONS

### Case 1

**FIGURE 1.**
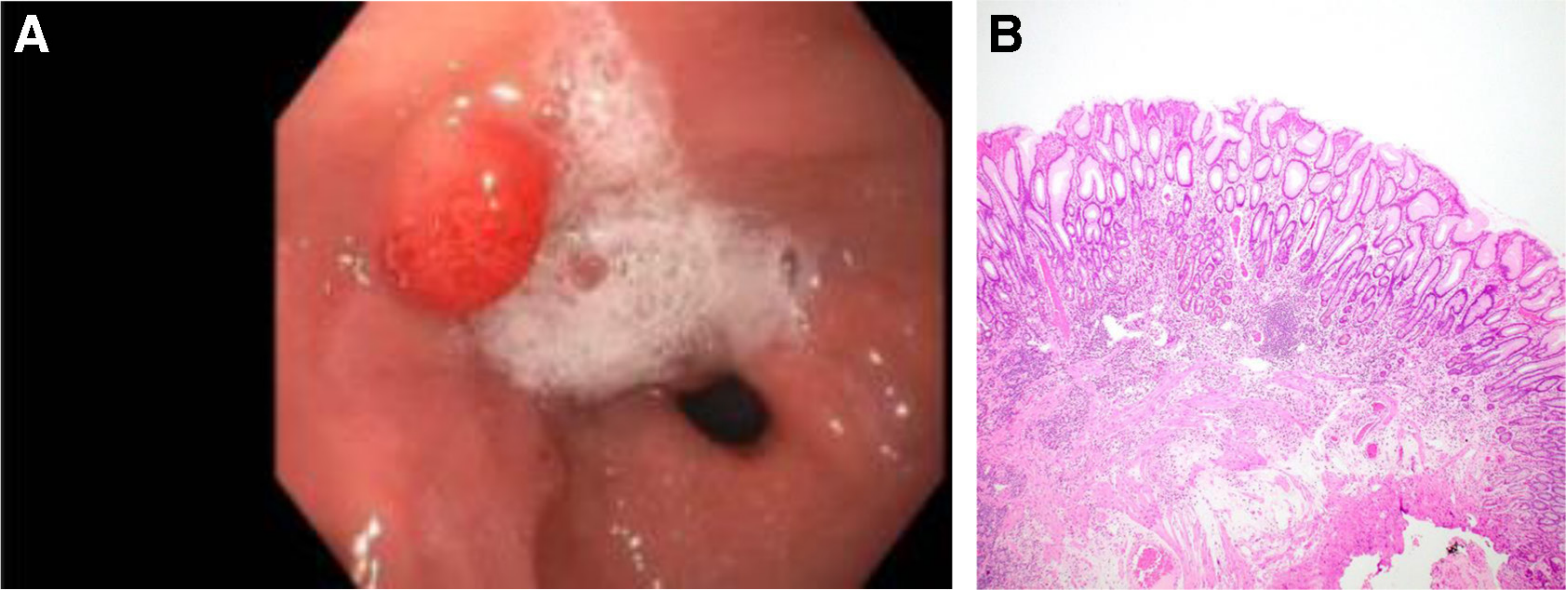
A) Endoscopy 12 month post treatment initiation: foveolar hyperplastic antral polyp. B) Histology 12 month post treatment initiation: foveolar hyperplastic polyp.

A 10-year-old male with SBS due to gastroschisis with 44 cm of residual small bowel length in continuity with a partial colon was dependent on parenteral and enteral nutrition. The patient was not receiving H2-receptor antagonist or proton pump inhibitor (PPI) therapy before teduglutide initiation and was receiving long-standing enteral iron supplementation. Upper endoscopy and colonoscopy were performed before initiation of teduglutide for evaluation of diffuse abdominal pain and diarrhea. Neither large nor small bowel polyps were identified. Stool evaluation for follow-up of his enteritis revealed negative fecal occult blood after 7 months of treatment. Per manufacturer recommendations, he underwent surveillance colonoscopy after 12 months of treatment, which was normal. An upper endoscopy was performed due to clinical concern for small bowel bacterial overgrowth showing a 10-mm gastric antrum sessile polyp (Fig. 1A). No *Helicobacter pylori* was seen on biopsies. Uncomplicated polypectomy was performed using saline injection-lift technique and hot snare. Pathology confirmed foveolar hyperplastic polyp and positive iron stain consistent with iron pill–associated mucosal injury (Fig. 1B). Teduglutide was continued. While the manufacturer’s recommendations indicate a colonoscopy every 5 years or earlier with new or unexplained Gastrointestinal (GI) bleeding, given the foveolar polyp found, yearly follow-up upper endoscopy and colonoscopy are planned.

### Case 2

**FIGURE 2.**
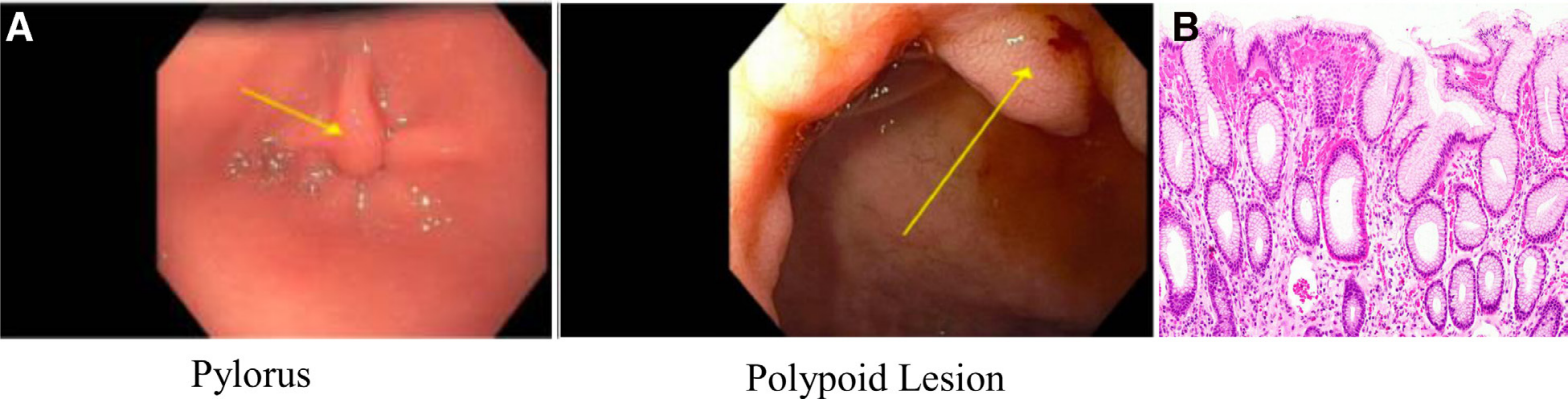
A) Endoscopy 15 month post treatment initiation: foveolar hyperplastic polyp with extension into duodenum. B) Histology 15 month post treatment initiation: foveolar hyperplastic polyp.

A 7-year-old male with SBS due to gastroschisis with 40 cm residual small bowel length in continuity with the mid-transverse colon was sustained on parenteral and enteral nutrition. His course was complicated by chronic small bowel ulceration. Upper endoscopy and colonoscopy before teduglutide initiation were performed for positive fecal occult blood showing chronic inactive gastritis and a perianastomotic ulceration with mildly active enteritis. He was then initiated on teduglutide and enteral anti-inflammatory therapy and continued treatment for small intestinal bacterial overgrowth. He was not receiving H2-receptor antagonist or PPI therapy before teduglutide. While the manufacturer recommends a colonoscopy 12 months after treatment initiation, he underwent colonoscopy at 15 months due to scheduling difficulties. The colonoscopy was normal. Given prior gastritis, an upper endoscopy was also performed at that time showing a prominent pyloric fold with polypoid extension into the duodenum. Biopsies of this lesion (Fig. 2A) were consistent with a foveolar hyperplastic polyp. Biopsies were otherwise normal, including no evidence of *Helicobacter pylori* (Fig. 2B). Teduglutide was continued. While the manufacturer recommends a colonoscopy every 5 years or earlier with new or unexplained GI bleeding, given the presence of a foveolar hyperplastic polyp, follow-up upper endoscopy and colonoscopy are planned.

## DISCUSSION

In trials of adult patients, colonic polyps are a known but rare sequela of teduglutide. As teduglutide use has continued beyond the initial study time frame, there have been increasing reports of both benign and malignant small bowel polyp development (10–12,14,15). In trials of pediatric patients, limited to 12 and 24 weeks, initial upper endoscopy and colonoscopies were not required. In addition, postexposure upper endoscopy and colonoscopy was not the standard of care (9,13,16). Thus, the incidence of intestinal polyp formation on teduglutide treatment in pediatric patients remains unknown. A recent retrospective review of adult patients with SBS showed increased small bowel polyp formation in 8 out of 35 patients (22.9%) on long-term teduglutide use. These included 4 in the duodenal bulb, 1 in the the duodenum, 1 in the ampulla, and 2 in the distal jejunum. Of these 8 polyps, 5 were foveolar hyperplastic lesions without dysplasia and 3 were identified as adenomas with low-grade dysplasia. Notably, none of the patients showed signs and symptoms concerning for a polyp (14).

In a current multicenter long-term observational study (https://www.clinicaltrials.gov; NCT04832087), patients with hemoccult-positive stools upon enrollment undergo a colonoscopy before treatment. Enrolled patients then undergo surveillance colonoscopy at 1 year of treatment. If patients have hemoccult-positive stools or are symptomatic, an upper GI endoscopy is also performed. Routine colonoscopies are planned every 5 years or sooner if clinically indicated or if stools are hemoccult positive. A recent study of adult patients with SBS showed the occurrence of both foveolar and dysplastic small bowel polyps in some patients on long-term teduglutide (14). We report 2 cases of foveolar polyps found in pediatric patients at 1 year of teduglutide treatment. At the time of endoscopy for case 1 and case 2, 15 and 24 patients had been on teduglutide for at least 1 year, respectively, and neither small bowel nor colonic polyps were identified outside of the 2 described reports.

Foveolar cells are mucus-producing columnar cells lining the gastric luminal surface. The presence of foveolar hyperplasia has been associated with superficial damage to the gastric mucosa seen with chronic use of nonsteroidal anti-inflammatory drugs, PPIs, bile exposure, and reactive changes associated with *Helicobacter pylori*. Each of these with the exception of PPIs cause a chronic caustic injury to the gastric epithelium stimulating a hyperplastic response. Chronic inhibition of gastric secretion by PPIs leads to enlarged parietal cells and hypergastrinemia. It is hypothesized that this hypergastrinemia stimulates the development of foveolar epithelial hyperplasia presenting as hyperplastic fundic gland gastric polyps or as white, flat elevated lesions (17).

In general, isolated foveolar hyperplasia has not been identified as a premalignant lesion. Foveolar hyperplasia found in fundic gland polyps in patients with familial adenomatous polyposis (FAP), however, has been shown to be pathogenically different from fundic gland polyps in non-FAP patients and are considered neoplastic changes (18). The connection between foveolar hyperplasia and development of dysplasia, though, remains poorly understood, and further work delineating the natural history of foveolar polyps in the context of teduglutide is important. In this report, neither patient has undergone genetic testing for polyposis syndromes, including FAP.

While foveolar hyperplasia is not inherently concerning for increased malignancy, the development of polyps in conjunction with a drug that stimulates epithelial proliferation merits further review (15). This is important given that in the initial trials of pediatric patients, treatment was discontinued at the 1-year mark (9,13). One possible mechanism for foveolar polyp formation with teduglutide is that teduglutide, similar to GLP2, decreases acid secretion and mimics chronic PPI use (17). Delayed antral motility was also seen in animal models with GLP2, suggesting decreased bile clearance from biliary reflux as another proposed mechanism for foveolar polyp development with exposure to teduglutide (19).

Colorectal polyp formation is a known sequalae of long-term teduglutide use based on data from trials with adult patients. The initial trials in pediatrics, however, did not include routine upper endoscopy or colonoscopy. A long-term observational study of pediatric use of teduglutide is currently underway, which could provide data on the incidence of small bowel and colorectal polyp formation in these patients. In addition to understanding the incidence of polyp development in patients on long-term teduglutide, genetic analysis of these polyps may be key to further characterizing the types of polyps, as well as their risk of malignancy. Given the limited data on foveolar polyps in teduglutide use, the clinical decision was made to follow the patients in this case report more closely with yearly upper endoscopy and colonoscopy. As we continue to understand the utility and sequelae of long-term teduglutide use, it will be important to identify if patients are at increased risk of colorectal and small bowel polyp formation as a direct consequence of GLP-2 analog use. In addition, understanding the types of polyps and the risk of progression to dysplasia will be key. Our cases along with recently published data of adult patients with SBS on teduglutide (14) may warrant revision of the drug and study protocols for inclusion of upper endoscopy, as well as more frequent endoscopic evaluations as the standard of care for patients on long-term teduglutide.

## ACKNOWLEDGMENTS

Informed consent for publication of these findings was obtained from the parents of both individuals. Specific identifying details were removed to protect patient anonymity.
